# Stimulation of Angiotensin Converting Enzyme 2: A Novel Treatment Strategy for Diabetic Nephropathy

**DOI:** 10.3389/fphys.2021.813012

**Published:** 2022-01-11

**Authors:** Haru Nomura, Sanjaya Kuruppu, Niwanthi W. Rajapakse

**Affiliations:** ^1^School of Biomedical Sciences, University of Queensland, Brisbane, QLD, Australia; ^2^Biomedicine Discovery Institute, Monash University, Melbourne, VIC, Australia

**Keywords:** diabetes, diabetic nephropathy, renin angiotensin aldosterone system (RAAS), angiotensin converting enzyme 2, angiotensin converting enzyme 2 stimulators

## Abstract

Despite current therapies for diabetic nephropathy, many patients continue to progress to end-stage renal disease requiring renal replacement therapy. While the precise mechanisms underlying diabetic nephropathy remain to be determined, it is well established that chronic activation of the renin angiotensin aldosterone system (RAAS) plays a substantial role in the pathogenesis of diabetic nephropathy. Angiotensin converting enzyme 2 (ACE2), the enzyme responsible for activating the reno-protective arm of the RAAS converts angiotensin (Ang) II into Ang 1-7 which exerts reno-protective effects. Chronic RAAS activation leads to kidney inflammation and fibrosis, and ultimately lead to end-stage kidney disease. Currently, angiotensin converting enzyme inhibitors and Ang II receptor blockers are approved for renal fibrosis and inflammation. Targeting the reno-protective arm of the RAAS should therefore, provide further treatment options for kidney fibrosis and inflammation. In this review, we examine how targeting the reno-protective arm of the RAAS can ameliorate kidney inflammation and fibrosis and rescue kidney function in diabetic nephropathy. We argue tissue ACE2 stimulation provides a unique and promising therapeutic approach for diabetic nephropathy.

## Introduction

The prevalence of diabetes mellitus continues to increase in both Australia and worldwide and it remains the number one cause of chronic kidney disease (Whiting et al., 2011; International Diabetes Federation, 2019; ANZDATA Registry, 2020). Despite the current treatments for diabetic nephropathy, patients progress to end-stage renal disease requiring renal replacement therapy (ANZDATA Registry, 2020). In addition to renin angiotensin aldosterone system (RAAS) blockade or SGLT2 inhibition, more therapies that reduce nephron loss and thus, halt the progression of diabetic nephropathy is required (McGrath and Edi, 2019; Perkovic et al., 2019). Chronic activation of the RAAS is a key driver of diabetic nephropathy, and as such, new approaches targeting the reno-protective arm of the RAAS can provide the basis for developing new treatments. Here, we review the evidence which indicate that targeting kidney tubular and glomerular angiotensin converting enzyme 2 (ACE2) can provide unique therapeutic options for diabetic nephropathy.

## Chronic Activation of the Renin Angiotensin Aldosterone System as a Main Driver of Diabetic Nephropathy

Chronic RAAS activation and consequent increase in oxidative stress, renal inflammation, and fibrosis are major contributors to the progression of diabetic nephropathy (Mezzano et al., 2003; Lakshmanan et al., 2011; Liu et al., 2011; Tojo et al., 2016; Wysocki et al., 2017; Zhao et al., 2018; Malek et al., 2019; Tang et al., 2019; Wang et al., 2019; Warren et al., 2019; Lu et al., 2020; Yang et al., 2020; de Alcantara Santos et al., 2021). Increased levels of the RAAS components have been detected in experimental diabetic nephropathy as well as in renal biopsies of patients with diabetic kidney disease (Mezzano et al., 2003; Lakshmanan et al., 2011; Liu et al., 2011). While ACE inhibitors and Ang II receptor blockers remain as the first line treatment for diabetic nephropathy, there is a need to develop new treatment approaches which can rescue kidney function, particularly kidney fibrosis (McGrath and Edi, 2019). In this context, enhancing the activity of the counter-regulatory arm of the RAAS can further reduce the effects of Ang II thereby, having anti-inflammatory and anti-fibrotic effects.

It is well established that fibrosis and inflammation in the kidney tubules play a substantial role in driving diabetic nephropathy (Lin et al., 2018). Significant increase in mRNA and protein levels of tumour growth factor-β1 (TGF-β1), plasminogen activator inhibitor-1 (PAI-1) and fibronectin was observed in the renal cortex of *db/db* mice (Zhou et al., 2014). Furthermore, augmented mRNA and protein levels of pro-inflammatory marker tumour necrosis factor-α (TNF-α) was observed in the renal cortex of *db/db* mice compared to control mice (Zhou et al., 2014). This was associated with increased urinary albumin excretion (Zhou et al., 2014). Administration of Ang 1-7 ameliorates diabetic kidney disease in a wide variety of experimental diabetic models, indicating that activation of the counter-regulatory arm of the RAAS is highly beneficial in diabetic nephropathy (Giani et al., 2012; Mori et al., 2014; Zhang et al., 2015; Cassis et al., 2019).

## Counter-Regulatory Arm of the Renin Angiotensin Aldosterone System Associated With Angiotensin Converting Enzyme 2/Ang 1-7/Mas Receptor

A new component of the RAAS was discovered over a decade ago, known as ACE2 (Donoghue et al., 2000). It is a zinc-dependent monocarboxypeptidase with 60% sequence similarity to ACE. ACE2 is mainly found in the heart, kidneys, testes and intestine (Tipnis et al., 2000). ACE2 plays a major role in the breakdown of Ang II and therefore, it is recognised as a negative regulator of the classical RAAS (Patel et al., 2014). While ACE2 is localised to the cell membrane, it can shed to produce a soluble form of the enzyme which is often detected in the urine of diabetic patients (Liang et al., 2015; Gutta et al., 2018). This is mediated by a disintegrin and metalloproteinase-17 (ADAM17). Hyperglycemia and Ang II enhances the activity of ADAM17 which can underpin increased levels of soluble ACE2 in the setting of diabetes (Salem et al., 2014; Xiao et al., 2014). Indeed, knockout of ADAM17 in the renal proximal tubules of streptozotocin (STZ)-induced diabetic mice showed reduced circulating ACE2 when compared with diabetic wild type control mice (Palau et al., 2021).

Cleavage of Ang II by ACE2 results in the formation of Ang 1-7 which then binds to the Mas receptor expressed in the kidney. Binding of Ang 1-7 to the Mas receptor exerts vasodilatory, anti-fibrotic and anti-inflammatory effects, thereby counteracting the pathological effects mediated by excess levels of Ang II (Donoghue et al., 2000). Ang 1-7 may also have the ability to modulate blood glucose levels as ACE2 knockout in wild type mice presented with significantly higher fasting glucose than the control wild type mice (Bindom and Lazartigues, 2009). ACE2 can also cleave Ang I to form Ang 1-7 *via* an intermediate peptide, Ang 1-9 (Donoghue et al., 2000). However, the catalytic efficiency of ACE2 with Ang II is 400-fold greater than that for Ang I (Vickers et al., 2002). Furthermore, assessment of kidney biopsies from living human transplant donors showed that ACE2 was the predominant enzyme responsible in the formation of Ang 1-7 (Domenig et al., 2016). Thus, in humans, the primary pathway for Ang 1-7 production is *via* the direct cleavage of Ang II. Ang 1-7 is also formed by direct cleavage of Ang I by neprilysin (NEP) or indirectly by cleaving Ang 1-9 (Domenig et al., 2016). It was reported in the kidney of normal mice that NEP is the major enzyme responsible in producing Ang 1-7 from Ang 1-9, rather than ACE2 (Domenig et al., 2016). NEP-mediated Ang 1-7 production was prominent in the renal cortical region, which was reduced in the presence of a NEP inhibitor, DL-Thiorphan (Domenig et al., 2016). Although Ang 1-7 formation by NEP is not the major route in humans, it is important to consider this pathway because dual inhibition of NEP and AT_1_R (LCZ696: sacubitril/valsartan) is considered as an alternative treatment approach in addition to RAAS inhibition for various cardiovascular diseases including heart failure (McMurray et al., 2014).

### Reno-Protective Effects of the Counter-Regulatory Arm of the Renin Angiotensin Aldosterone System

Modulating the activity and/or expression of ACE2 is a promising therapeutic approach for diabetic nephropathy due to the ability of ACE2 to both degrade Ang II and generate Ang 1-7 (Oudit et al., 2010; Shiota et al., 2010; Liu et al., 2011). Administration of recombinant human ACE2 (rhACE2) in diabetic Akita mice for 4 weeks significantly reduced renal fibrosis, glomerular hypertrophy, and glomerular basement membrane thickness (Oudit et al., 2010). Adenovirus-mediated ACE2 overexpression in STZ-induced diabetic rats resulted in significant downregulation in pro-fibrotic TGF-β1 and collagen IV expression (Liu et al., 2011). This coincided with significant reductions in albuminuria, glomerulosclerosis and oxidative stress (Liu et al., 2011). These reno-protective effects of ACE2 overexpression were similar to that seen with ACE inhibitor treatment (benazepril) (Liu et al., 2011). Similarly, podocyte-specific overexpression of ACE2 in STZ-induced diabetic mice resulted in reduced levels of mesangial expansion and glomerular hypertrophy, further supporting the reno-protective effects of ACE2 (Nadarajah et al., 2012).

On the other hand, ACE2-knockout in STZ-induced diabetic mice demonstrated accelerated glomerulosclerosis, tubular injury, interstitial fibrosis, podocyte apoptosis and increased serum creatinine levels (Shiota et al., 2010). Pharmacological inhibition of ACE2 using MLN-4760 in *db/db* mice and in STZ-induced diabetic mice resulted in increased fibronectin and collagen deposition in the glomerulus and tubulointerstitial area, in addition to mesangial matrix expansion (Ye et al., 2006; Soler et al., 2007). Collectively, results from these studies indicate that decreased levels of ACE2 lead to kidney injury in diabetes.

## Angiotensin Converting Enzyme 2 Distribution in the Kidney Vasculature

In renal biopsies from healthy human subjects, ACE2 was found in the smooth muscle cells of interlobular arteries (Lely et al., 2004). Additionally, ACE2 expression was evident in the endothelium of interlobular arteries and in small to medium-sized veins in the kidney of these subjects (Lely et al., 2004). Within the glomerulus of these subjects, ACE2 expression was observed in the glomerular visceral and parietal epithelial cells (Lely et al., 2004). ACE2 expression was absent in mesangial cells and glomerular endothelial cells in human subjects (Hamming et al., 2004; Lely et al., 2004). In the mouse renal vasculature, ACE2 was only expressed in the vascular smooth muscle cells of the renal arterioles and its expression was absent in the endothelial cells (Soler et al., 2009). ACE2 was also found in arcuate arteries and interlobular arteries in mice (Soler et al., 2009). In rats, ACE2 expression was evident in both vascular smooth muscle cells and endothelial cells of large arteries and afferent arterioles, and in the vasa recta (Li et al., 2005; Kamilic et al., 2010). Within the glomeruli of both mice and rats, ACE2 expression was absent in the endothelial cells, which is consistent with that seen in humans (Ye et al., 2006; Kamilic et al., 2010). In mice, ACE2 was found to be expressed on the podocyte foot processes, body and slit diaphragm of the podocyte, and mesangial cells to a lesser extent (Ye et al., 2006). ACE2 expression was observed in the glomerular visceral and parietal epithelial cells while no expression was detected in the mesangial cells of the rat glomeruli (Kamilic et al., 2010).

## Angiotensin Converting Enzyme 2 Distribution in the Kidney Tubules

In both human and rodent kidneys, ACE2 is highly abundant in the apical membrane of the proximal tubule (Hamming et al., 2004; Lely et al., 2004; Ye et al., 2004, 2006). Single-cell RNA sequencing analysis of 15 normal human kidney samples revealed high abundance of ACE2 in the proximal convoluted and straight tubules, further supporting the localization of ACE2 in the proximal tubules (Pan et al., 2020). High expression of ACE2 in this section of the nephron likely negates the harmful effects arising from the high concentration of Ang II evident in the lumen of the proximal tubules in humans (Li et al., 2005). In rats, ACE2 protein and mRNA expressions were evident throughout the nephron except in the medullary thick ascending limb (Li et al., 2005). More specifically, ACE2 expression was found in the proximal tubules (convoluted and straight), Loop of Henle (outer medullary thin limb and cortical thick ascending limb), distal tubules, and collecting ducts (Li et al., 2005). In line with the expression of ACE2, significant generation of Ang 1-7 was observed in the proximal straight tubules of normal rats (Li et al., 2005). Intracellular expression of ACE2 was also observed in proximal tubules, distal tubules and collecting ducts in normal rat and human kidneys (Hamming et al., 2004; Lely et al., 2004; Li et al., 2005; Joyner et al., 2007).

## Changes in the Classical and Reno-Protective Arm of the Renin Angiotensin Aldosterone System in Diabetic Nephropathy

Changes in ACE2 expression and activity that occur in diabetic nephropathy have been investigated intensively in both pre-clinical models and in patients with diabetic nephropathy (Wysocki et al., 2006; Reich et al., 2008). In hypertensive mRen2.Lewis rats with STZ-induced early-onset diabetes, the levels of circulating ACE2 in serum was increased in early stages of diabetes (Yamaleyeva et al., 2012). Increased ACE2 activity and protein levels were also observed in the renal cortical region of both *db/db* mice and STZ-induced diabetic mice at early stages of the disease, while observing no change in its mRNA levels (Wysocki et al., 2006). On the other hand, downregulation in ACE mRNA levels, protein expression and activity were observed in *db/db* mice and in STZ-induced diabetic mice (Ye et al., 2004; Wysocki et al., 2006). These opposing changes in ACE and ACE2 expression and/or activity may be a possible mechanism to compensate for the chronic activation of the RAAS and accumulation of Ang II in the kidney (Wysocki et al., 2006).

While increased activity and expression of ACE2 were reported in early stages of diabetes, downregulation in ACE2 expression has been observed in both pre-clinical studies and in patients with advanced stages of diabetic nephropathy (Tikellis et al., 2003; Reich et al., 2008). Reduced ACE2 expression has been reported in renal tubules and glomeruli of rodent diabetic animals (Tikellis et al., 2003). Consistent with these pre-clinical studies, ACE2 mRNA expression was significantly reduced in proximal tubules and in the glomerular compartment of renal biopsies obtained from patients with diabetic nephropathy (Reich et al., 2008). Moreover, in these patients, expression of ACE mRNA was significantly greater in both proximal tubules and glomeruli when compared to healthy control subjects (Konoshita et al., 2006; Reich et al., 2008). These findings suggest that the increase in ACE2 activity and expression observed in early stages of diabetes gradually decreases with disease progression (Tikellis et al., 2003; Reich et al., 2008).

## Techniques to Measure Angiotensin Converting Enzyme 2 Activity

Since ACE2 activity changes with disease progression in diabetes, it is critical to develop robust techniques to measure its activity in biological samples and tissues. Fluorometric ACE2 activity assay kits are commercially available but the accuracy of these tests in measuring tissue and plasma ACE2 activity remains to be validated. There are recent developments in novel assays to measure ACE2 activity in biological fluids using hydrolysis of an intramolecularly quenched fluorogenic ACE2 substrate (Xiao and Burns, 2017). Such assays can be the basis for development of more robust and accurate commercially available kits in future which can be widely used in experimental and clinical settings. Mass spectrometry-based methods are also available for measurement of ACE2 activity in the experimental setting (Elased et al., 2006). Of note, RAAS fingerprint using liquid chromatography-mass spectrometry (LC-MS)/MS technique is a gold standard technique to measure components of the RAAS and should be employed in quantifying ACE2 activity in diabetes (van Rooyen et al., 2016).

## Approaches to Increase the Effects of Angiotensin Converting Enzyme 2 in Diabetic Nephropathy

### Recombinant Human Angiotensin Converting Enzyme 2 Administration

One approach to enhance the reno-protective effects of ACE2 is by exogenous administration of rhACE2. Administration of rhACE2 has progressed into phase I clinical trials (Haschke et al., 2013). In healthy human subjects, single dose of rhACE2 administration (100, 200, 400, 800, and 1200 μg/kg) significantly reduced plasma Ang II levels (Haschke et al., 2013). Corresponding increase in plasma Ang 1-7 levels were also observed following administration of rhACE2 at doses of 100 and 200 μg/kg (Haschke et al., 2013). However, there was no difference in plasma Ang 1-7 levels in subjects receiving doses higher than 400 μg/kg of rhACE2 (Haschke et al., 2013). Similar effects were observed in normal healthy subjects receiving daily consecutive administration of rhACE2 (400 μg/kg for 3 or 6 days) (Haschke et al., 2013). It is important to note that rhACE2 has not been tested in patients with diabetic nephropathy.

In diabetic Akita mice administered rhACE2 for 4 weeks, plasma and renal cortical levels of Ang II were significantly lower than that of control diabetic mice (Oudit et al., 2010). In line with this finding, Ang 1-7 levels in the renal cortex was significantly greater than in control diabetic mice (Oudit et al., 2010). In these mice, urinary albumin excretion rate was significantly reduced compared to control diabetic Akita mice (Oudit et al., 2010). Administration of rhACE2 (0.1, 1 or 5 mg/kg/day) to healthy control mice for 3 consecutive days tended to reduce plasma Ang II levels and increase plasma Ang 1-7 levels, however, no significant effects on ACE2 activity in the renal cortex was observed (Wysocki et al., 2010). This lack of effect at the tissue level may be explained by the limited capillary permeability of a healthy glomerulus which limits the filtration of large proteins such as rhACE2 and thus, fail to reach renal tubular cells (Haschke et al., 2013; Wysocki et al., 2017). Therefore, rhACE2 as a therapy may only be effective in advanced diabetic nephropathy where patients experience overt proteinuria due to significantly injured glomerular filtration barrier, allowing rhACE2 to filter into the tubules (Wysocki et al., 2017). In the setting of advanced diabetic nephropathy, where local ACE2 levels are reduced and chronic activation of the RAAS is observed, it is crucial to target endogenous renal ACE2 to achieve optimum therapeutic benefit (Wysocki et al., 2010, 2017).

### Ang 1-7-Based Therapy

Ang 1-7 have demonstrated reno-protective effects in the setting of diabetic nephropathy (Mori et al., 2014). Chronic Ang 1-7 administration in experimental diabetes demonstrated improved pathophysiology of diabetic nephropathy (Mori et al., 2014). However, Ang 1-7 has a short half-life of 0.5 h and to overcome this, stable versions of Ang 1-7 have been developed such as AVE 0991 and cyclised Ang 1-7 (Wiemer et al., 2002; Kluskens et al., 2009). Cyclic Ang 1-7 exerts reno protection in BTBR *ob/ob* diabetic mice and also improves blood glucose levels in experimental diabetes (Cassis et al., 2019; Kuipers et al., 2019). However, to date, none of the stabilised versions of Ang 1-7 have progressed into the clinic, highlighting the challenges in translating Ang 1-7-based therapies to the clinic (Touyz and Montezano, 2018). Alternatively, targeting endogenous tissue ACE2 is a unique approach to increase Ang 1-7 levels. Stimulation of renal tubular ACE2 provides a unique approach to target tubular fibrosis and inflammation.

### Angiotensin Converting Enzyme 2 Stimulators Provide a Unique Approach to Target Tubular and Glomerular Angiotensin Converting Enzyme 2

Previous studies have reported the identification of two molecules, xanthenone and diminazene aceturate (DIZE), as ACE2 stimulators (Hernández Prada José et al., 2008; Kulemina and Ostrov, 2011). It was initially reported that xanthenone can decrease blood pressure, improve cardiac function, and reverse cardiac and renal fibrosis in spontaneously hypertensive rats (Hernández Prada José et al., 2008). However, it was later reported that these protective effects of xanthenone was independent of ACE2 stimulation (Haber Philipp et al., 2014). Similarly, DIZE was initially reported to reduce hypertension, and cardiac and renal fibrosis *in vivo*. However, its pharmacological effects were also found to be independent of ACE2 stimulation (Haber Philipp et al., 2014). Furthermore, a high concentration of DIZE (100 μM) inhibited the activity of ACE2 (Haber Philipp et al., 2014).

**FIGURE 1:**
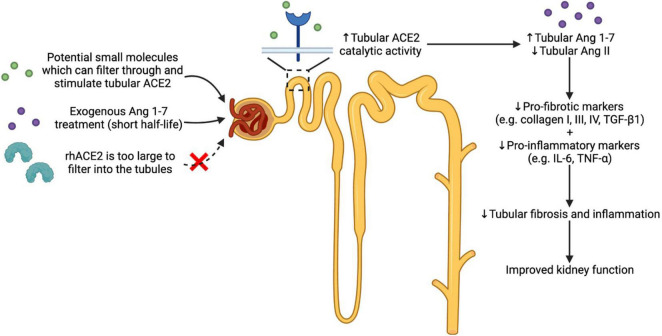
Effects of theoretical use of small molecules which activate the reno-protective arm of the RAAS (ACE2/Ang 1-7/Mas receptor axis). Activating the ACE2/Ang 1-7/Mas receptor axis is one approach in treating diabetic nephropathy where chronic activation of the classical RAAS is observed. ACE2 stimulation using potential small molecules which can filter into the renal tubules can increase the catalytic activity of tubular ACE2. This in turn can enhance the production of Ang 1-7 and degradation of Ang II, thereby reducing renal inflammation and fibrosis, leading to improved kidney function. Ang 1-7-based therapy is another approach in activating the reno-protective arm of the RAAS. However, the short half-life of Ang 1-7 is a major drawback, making it challenging to translate Ang 1-7-based therapy to the clinic. Additionally, large proteins such as rhACE2 cannot reach the renal tubular cells due to the limited capillary permeability. Abbreviations: RAAS: renin angiotensin aldosterone system; ACE2: angiotensin converting enzyme 2; Ang: angiotensin; rhACE2: recombinant human angiotensin converting enzyme 2; TGF-β1: tumour growth factor-β1; IL-6: interleukin-6; TNF-α: tumour necrosis factor-α.

Hence to date, there is no ACE2 stimulator that has progressed into the clinic, indicating that there is an unmet need for clinically useful ACE2 stimulators. An effective small molecule ACE2 stimulator will promote protective effects by activating the ACE2/Ang 1-7/Mas receptor axis of the RAAS, thereby shifting the balance from Ang II to Ang 1-7 (Reich et al., 2008). The strength of stimulating the catalytic activity of tubular ACE2 is the ability to both produce tissue Ang 1-7 and degrade excess Ang II, thereby reducing tubular fibrosis and inflammation in the setting of diabetes (Figure 1). It is important to note that Ang 1-7-based therapies cannot alter Ang II levels. Moreover, rhACE2 treatment is unable to cross the glomerular filtration barrier to reach kidney tubular cells, limiting its use for tubular injury in diabetic nephropathy.

## Conclusion

The prevalence of diabetic nephropathy and the consequent progression to end-stage renal disease is increasing worldwide. Current therapies and management strategies mainly aim to delay disease progression but are unable to halt or restore kidney function. Chronic activation of the RAAS plays a central role in inducing renal inflammation and fibrosis, ultimately leading to decline in kidney function. Compounds which increase ACE2 catalytic activity provides a unique approach to target tubular and glomerular ACE2, opening up new treatment approaches for diabetic nephropathy. There is the need to develop new ACE2 stimulators with clinical translational potential.

## Author Contributions

HN, SK, and NR equally and directly contributed to the manuscript. All authors contributed to the article and approved the submitted version.

## Conflict of Interest

The authors declare that the research was conducted in the absence of any commercial or financial relationships that could be construed as a potential conflict of interest.

## Publisher’s Note

All claims expressed in this article are solely those of the authors and do not necessarily represent those of their affiliated organizations, or those of the publisher, the editors and the reviewers. Any product that may be evaluated in this article, or claim that may be made by its manufacturer, is not guaranteed or endorsed by the publisher.
